# Transcriptional Regulation of Atp-Dependent Chromatin Remodeling Factors: Smarcal1 and Brg1 Mutually Co-Regulate Each Other

**DOI:** 10.1038/srep20532

**Published:** 2016-02-04

**Authors:** Dominic Thangminlen Haokip, Isha Goel, Vijendra Arya, Tapan Sharma, Reshma Kumari, Rashmi Priya, Manpreet Singh, Rohini Muthuswami

**Affiliations:** 1 Chromatin Remodeling Laboratory, School of Life Sciences, Jawaharlal Nehru University, New Delhi 110067.

## Abstract

The ATP-dependent chromatin remodeling factors regulate gene expression. However, it is not known whether these factors regulate each other. Given the ability of these factors to regulate the accessibility of DNA to transcription factors, we postulate that one ATP-dependent chromatin remodeling factor should be able to regulate the transcription of another ATP-dependent chromatin remodeling factor. In this paper, we show that BRG1 and SMARCAL1, both members of the ATP-dependent chromatin remodeling protein family, regulate each other. BRG1 binds to the *SMARCAL1* promoter, while SMARCAL1 binds to the *brg1* promoter. During DNA damage, the occupancy of SMARCAL1 on the *brg1* promoter increases coinciding with an increase in BRG1 occupancy on the *SMARCAL1* promoter, leading to increased *brg1* and *SMARCAL1* transcripts respectively. This is the first report of two ATP-dependent chromatin remodeling factors regulating each other.

ATP-dependent chromatin remodeling factors are essential components for creating epigenetic states^1^. These proteins utilize the energy released by hydrolysis of ATP to reposition nucleosomes, thus, creating euchromatin and heterochromatin states. The ATP-dependent chromatin remodeling proteins were initially isolated from *S. cerevisiae* and, based on sequence homology, have been classified into 24 sub-families^2^. These proteins have been shown to play an important role not only in transcription regulation but also in DNA repair and replication^3^,^4^,^5^.

Given their central role in DNA processes it is expected that these proteins would be regulated^6^. It is well-known that the regulation of these proteins happens post-translationally at multiple levels. Studies have shown that BRG1 and BRM, mammalian homologues of the yeast Snf2 protein, are phosphorylated during mitosis, which results in their exclusion from the mitotic DNA^7^. The activity of BRM is regulated not only by phosphorylation but also by acetylation, a modification required for its interaction with Rb^7^,^8^. In contrast, Mi-2 is present as phospho-protein throughout the cell cycle and phosphorylation is important for limiting the ATPase activity of the protein^9^. Further, many of the ATP-dependent chromatin remodeling factors are components of multi-subunit complexes and thus, their activity can also be regulated by the subunit components. For example, BAF155, BAF170 and INI have been shown to stimulate the ATPase activity of BRG1 and BRM in the SWI/SNF complexes^10^. Similarly, in the ACF1 complex, ACF1 has been shown to stimulate the activity of ISWI, an ATP-dependent chromatin remodeling factor involved in various DNA processes^11^. Another level of regulation involves alterations in the subunit composition of these multi-subunit complexes. The mammalian cells contain BAF and PBAF complexes. BAF complex can contain either BRG1 or BRM as the ATPase subunit, while PBAF complex contains only BRG1^12^,^13^. Further, the subunit compositions of BRG1 and BRM containing multi-subunit complexes can change through the developmental stages^14^. Finally, the ATP-dependent chromatin remodeling factors are usually recruited to their targets via histone modification, and thus, the histone modifying enzymes as well as incorporation of histone variants can also regulate the function of the SWI/SNF proteins^15^,^16^.

However, there are no reports as yet of whether one ATP-dependent chromatin remodeling protein can regulate the transcription of another ATP-dependent chromatin remodeling protein, though it is logical to expect the existence of such a phenomenon within a cell, as gene expression requires nucleosome remodeling.

In this paper, we show that SMARCAL1 and BRG1, two ATP-dependent chromatin remodeling proteins, regulate each other when HeLa cells are treated with doxorubicin. Localization as well as transcript level of SMARCAL1 was found to alter during different cell cycle stages as observed by immunocytochemistry and quantitative real-time RT-PCR respectively. Treatment of cells with doxorubicin, a DNA damage inducing agent, resulted in increased *SMARCAL1* transcripts that correlated with increased protein levels. Analysis of *SMARCAL1* promoter showed the presence of a positive regulatory region upstream of the putative transcription start site, where chromatin immunoprecipitation (ChIP) assays revealed the presence of BRG1. Downregulation of BRG1 resulted in decreased occupancy of H3K9Ac and RNA polymerase II (RNAPII) on *SMARCAL1* promoter resulting in reduced *SMARCAL1* transcript. Concomitantly, in SMARCAL1 downregulated cells, the *brg1* transcript was found to be downregulated. ChIP assays confirmed that SMARCAL1 was localized on the *brg1* promoter. The occupancy of SMARCAL1 on the *brg1* promoter increased when cells were treated with DNA damaging agent. Biochemical assays showed that SMARCAL1 can bind to the *brg1* promoter, hydrolyze ATP and use the energy to induce conformational change in the promoter. We postulate that BRG1 and SMARCAL1 possibly regulate each other as both are required for double-stranded DNA break repair^17^,^18^.

## Results

### SMARCAL1 is present in both cytoplasm and nucleus

To get an insight into the cellular functions of SMARCAL1, we began by exploring the cellular localization of the protein. We analyzed the localization of SMARCAL1 in asynchronous population of cells using custom-synthesized polyclonal antibody, raised against the unique N-terminus region of the protein^19^. Studies performed using different cell lines revealed that the protein was present both in the cytoplasm and the nucleus (Supplementary Fig. S1). The same localization pattern was observed when monoclonal antibodies raised against the bovine SMARCAL1 were used^20^ (Supplementary Fig. S1).

To further confirm the localization pattern, SMARCAL1 cDNA was cloned into LAP-Zeo vector and transiently transfected into HeLa cells. This experiment enabled us to observe populations of cells with both nuclear and cytoplasmic localization as well as cells with predominantly nuclear localized SMARCAL1 (Supplementary Fig. S1).

### Nuclear localization of SMARCAL1 is cell cycle dependent

**Figure 1:**
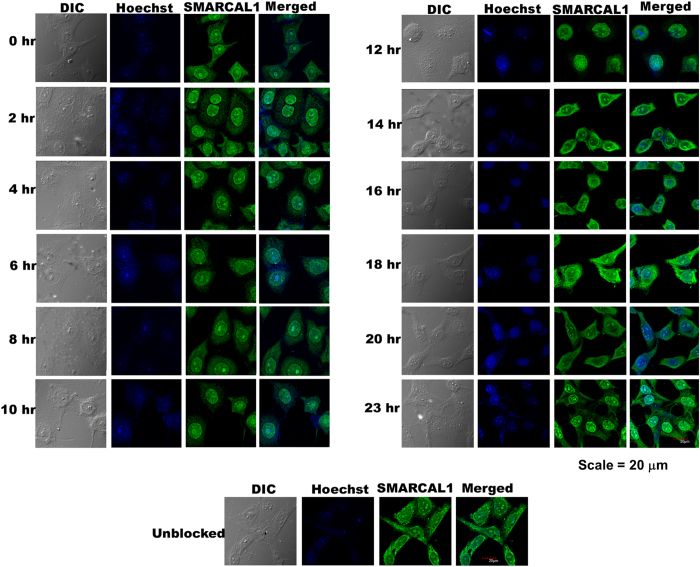
Cell cycle dependent localization of SMARCAL1. HeLa cells were synchronized using double thymidine block as described in materials and methods. Cells were released at various time-points and analyzed for SMARCAL1 localization using polyclonal antibody against the endogenous protein. The nucleus was stained using Hoechst 33342.

We next asked whether the cellular localization of SMARCAL1 changed as a function of different cell cycle stages. HeLa cells were synchronized using double thymidine block and the cells, after release, were analyzed by immunofluorescence (Fig. 1; Supplementary Fig. S2 and S3; Supplementary Table S1). The cells released at G1/S phase (0 hours) showed predominant nuclear localization of the protein along with partial cytoplasmic localization and this pattern was maintained as cell cycle proceeded through S phase (4 hours) to G2 and M phase (10–12 hours). However, the prominent nuclear localization was lost as the cells traversed through the 14 hours to 20 hours stage coinciding with the G1 phase. The pronounced nuclear localization was again observed at the 23 hours stage that marks the restart of a fresh cell cycle. These results, therefore, showed that the localization of SMARCAL1 was altered during different cell cycle stages and that the protein was maximally localized in the nucleus only after the G1/S transition phase up to the end of M phase.

### The levels of SMARCAL1 vary as a function of DNA damage

**Figure 2:**
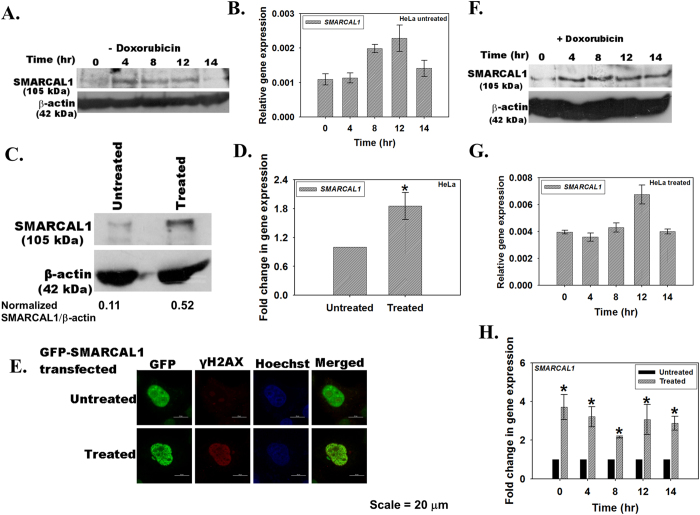
The transcript and protein levels of SMARCAL1 vary as a function of DNA damage. (**A**) HeLa cells were synchronized using double thymidine block and collected every two hours after release from block. SMARCAL1 expression was analyzed by western blot using polyclonal antibody against the protein. (**B**) The transcript levels in the synchronized cells after release from the double thymidine block were analyzed by quantitative real-time RT-PCR at time points indicated. (**C**) Asynchronous population of HeLa cells were treated with 2 μM doxorubicin for 10 minutes and SMARCAL1 levels were compared to the untreated cells by western blot using polyclonal antibody against SMARCAL1. The quantitation was done using ImageJ software. (**D**) The transcript levels in untreated and doxorubicin treated cells were compared using quantitative real-time RT-PCR (p value  < 0.05). (**E**) GFP-SMARCAL1 was transiently transfected into HeLa cells and treated with doxorubicin for 10 minutes. The localization of SMARCAL1 and γH2AX was probed using monoclonal anti-GFP antibody and anti-γH2AX antibody. The secondary antibodies were conjugated to FITC and TRITC respectively. Nucleus was stained using Hoechst 33342. Pearson’s coefficient for SMARCAL1-γH2AX co-localization in treated cells was 0.44 ± 0.17 while in untreated cells it was −0.012 ± 0.15. >10 cells were counted in this experiment. (**F**) HeLa cells were synchronized using double thymidine block and cells were harvested every two hours after release from the block. Prior to harvesting, the cells were treated for 10 minutes with 2 μM doxorubicin. The levels of SMARCAL1 were analyzed by western blot using polyclonal antibody against the protein. (**G**) The transcript levels in these cells were analyzed by quantitative real-time RT-PCR. (**H**) Comparison of the transcript levels in untreated and doxorubicin treated cells. The difference between untreated and doxorubicin treated cells was significant with p values  < 0.05 for each of the time point. In all these experiments, GAPDH was used as the internal control. Uncropped western blots are provided in Supplementary Fig. S11.

As SMARCAL1 localization altered through the cell cycle stages, we sought to determine whether the levels of SMARCAL1 also changed as a function of cell cycle. HeLa cells were synchronized using double thymidine block and the levels of SMARCAL1 were determined every four hours after release using quantitative real-time RT-PCR and western blot. As shown in Fig. 2A, the expression of SMARCAL1 appeared to increase 4 hours post-release coinciding with its proposed function in DNA repair during S phase^21^,^22^. However, the changes in SMARCAL1 expression during different cell cycle stages both at protein and RNA level were not significant (Fig. 2A,B).

As SMARCAL1 is known to play a role in DNA damage, therefore, we next asked whether the levels of SMARCAL1 changed when cells were treated with DNA damaging agents^17^,^21^,^22^. Asynchronous population of HeLa cells were treated with 2 μM doxorubicin for 10 minutes, which induces double-stranded DNA breaks (Supplementary Fig. S4), and SMARCAL1 expression was estimated using both quantitative real-time RT-PCR and western blot. As shown in Fig. 2C,D, treatment of cells with doxorubicin resulted in a 2-fold increase in SMARCAL1 levels. This correlated with the formation of SMARCAL1 foci in the nucleus (Fig. 2E).

We also studied the effect of doxorubicin treatment during different cell cycle stages. HeLa cells, synchronized using double thymidine block, were collected every four hours after release. Prior to collection, the cells were treated with doxorubicin for ten minutes. As shown in Fig. 2F,G, after doxorubicin treatment the expression of SMARCAL1 as a function of cell cycle did not change appreciably. However, comparison of the transcript levels in the treated and untreated sample at each time point along the different cell cycle stages showed significant upregulation, indicating that SMARCAL1 expression was induced when DNA was damaged by doxorubicin treatment (Fig. 2H).

### Human *SMARCAL1* promoter possesses two positive regulatory elements and a repressor element upstream of the transcription initiation site

To understand transcriptional regulation of *SMARCAL1* gene when DNA damage was induced, the promoter element of the gene was analyzed for its regulatory sequences. Bioinformatic analysis using Softberry promoter prediction software (www.softberry.com) as well as information from Huang *et al*.^23^ showed that a putative promoter sequence was present upstream of the translation start sequence and the promoter region was enriched in CpG islands.

A region spanning the putative transcription initiator sequence (TSS) and 2.1 kb upstream was amplified using gene specific primers and cloned into TA vector. Deletion constructs were made as indicated in Supplementary Fig. S5 and cloned into pGL3 basic vector. HeLa cells were transfected with these constructs and luciferase reporter activity was measured 36 hours post-transfection (Supplementary Fig. S5). The reporter activity results indicated the presence of two positive regulatory elements (Enh1 and Enh2) around 1.8 kb and 1.3 kb upstream respectively and a repressor element present around 700 bp upstream of the TSS.

The function of the positive regulatory elements and the repressor elements was confirmed by cloning these sequences into pGL3-promoter vector and assaying for the reporter luciferase activity 36 hr after transfecting them into HeLa cells (Supplementary Fig. S5). The putative positive regulatory sequences, Enh1 and Enh2, were able to activate transcription of the reporter gene while the putative repressor sequence, Rep, repressed the transcription of luciferase gene, confirming their respective functions. Further, as shown in Supplementary Fig. S5, Rep does not act as a promoter; however we did observe minimal promoter activity with both Enh1 and Enh2. As CpG island containing promoters show dispersive initiation, it is possible that Enh1 and Enh2 possess cryptic initiation sites. Further, Sp1 transcription factor is known to regulate transcription from CpG containing promoters and ChIP analysis confirmed that Sp1 does bind to the *SMARCAL1* promoter (Supplementary Fig. S5).

### BRG1 is present at the positive regulatory element of *SMARCAL1* promoter

We next investigated whether ATP-dependent chromatin remodeling proteins were required for SMARCAL1 expression. We chose BRG1 as it is a transcriptional modulator and has been shown to regulate expression of many genes^3^. ChIP analysis showed that BRG1 was indeed present at Enh1 and Enh2 region of *SMARCAL1* promoter in untreated HeLa cells (Supplementary Fig. S6). In addition, BRG1 was also found to occupy the region around the putative transcription start site (Supplementary Fig. S6). ChIP analysis also showed that both RNAPII and H3K9Ac were present on Enh2 as well as initiator region along with BRG1 in untreated HeLa cells (Supplementary Fig. S6).

**Figure 3:**
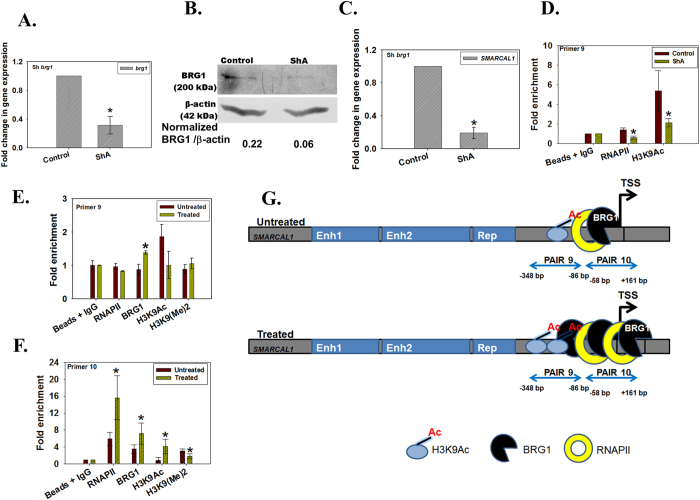
BRG1 positively regulates *SMARCAL1*. (**A**) *brg1* transcript levels were estimated by quantitative real-time RT-PCR in *brg1* downregulated ShA clone. The difference was significant for all with p values  < 0.05. (**B**) Western blot confirming the downregulation of BRG1 in ShA clone. (**C**) *SMARCAL1* transcript levels were estimated by quantitative real-time RT-PCR in *brg1* downregulated clones ShA. The difference was significant with p value  < 0.05. (**D**) ChIP analysis was performed to determine the occupancy of RNAPII and H3K9Ac at *SMARCAL1* promoter in *brg1* downregulated ShA cells. The difference was significant for both RNAPII and H3K9Ac with p values  < 0.05. (**E**) Occupancy of RNAPII, BRG1, H3K9Ac, H3K9(Me)2 on *SMARCAL1* promoter was analyzed using primer pair 9 by quantitative real-time RT-PCR after treatment with 2 μM doxorubicin for 10 minutes. The difference was significant for all with p values  < 0.05. (**F**) Occupancy of RNAPII, BRG1, H3K9Ac, H3K9(Me)2 on *SMARCAL1* promoter was analyzed using primer pair 10 by quantitative real-time PCR after treatment with 2 μM doxorubicin for 10 minutes. The difference was significant for all with p values  < 0.05. In all these experiments, GAPDH was used as the internal control. (**G**) Model explaining the regulation of *SMARCAL1* by BRG1 when cells are treated with doxorubicin. The uncropped western blot is provided in Supplementary Fig. S12.

To confirm BRG1 regulates *SMARCAL1* expression, we downregulated *brg1* in HeLa cells using shRNA and isolated a clone ShA. Using quantitative real-time RT-PCR and western blot we confirmed that *brg1* was downregulated (Fig. 3A,B). As expected, *SMARCAL1* was also found to be downregulated in this clone (Fig. 3C). Further, ChIP analysis showed that the RNAPII and H3K9Ac occupancy was significantly reduced at the primer 9 region of *SMARCAL1* promoter (Fig. 3D).

Finally, we examined the occupancy of BRG1 on *SMARCAL1* promoter when cells were treated with 2 μM doxorubicin for 10 minutes and found that the occupancy of BRG1, was increased both around the transcription start site as well as ~200 bp upstream of the transcription start site correlating with elevated SMARCAL1 levels leading us to conclude that BRG1 regulates *SMARCAL1* transcription (Fig. 3E–G). Further, the occupancy of RNAPII and H3K9Ac was increased around the transcription start site, again correlating with upregulated expression of *SMARCAL1* (Fig. 3F,G).

### BRG1 expression is upregulated when DNA is damaged

We hypothesized that if BRG1 regulates SMARCAL1, then its levels too should be regulated during DNA damage. To test the hypothesis, HeLa cells were treated with 2 μM doxorubicin for 10 minutes and the expression of BRG1 was analyzed by quantitative real-time RT-PCR and western blot. As shown in Supplementary Fig. S7, BRG1 expression was upregulated after doxorubicin treatment. Comparative analysis showed that the levels of both SMARCAL1 and BRG1 increased approximately 2-fold upon doxorubicin treatment, indicating that both these proteins are upregulated when DNA is damaged (Supplementary Fig. S7).

We also found that BRG1 expression was upregulated at different cell cycle stages in the presence of doxorubicin as compared to that in the absence of doxorubicin in a manner reminiscent of SMARCAL1 levels (Supplementary Fig. S7).

### SMARCAL1 regulates BRG1 expression

**Figure 4:**
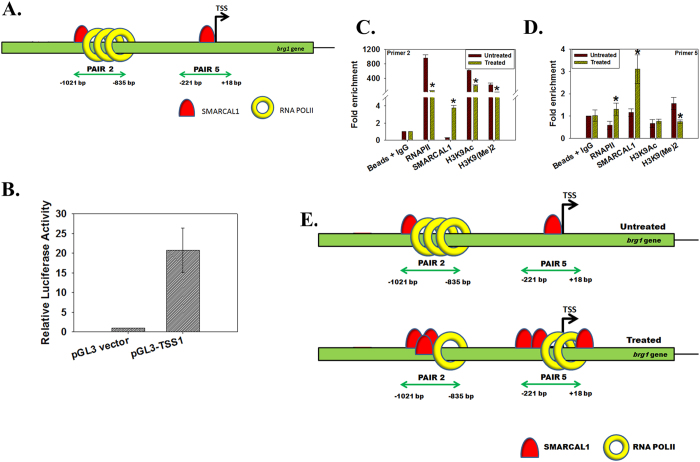
SMARCAL1 positively regulates *brg1.* (**A**) The architecture of *brg1* promoter with RNAPII and SMARCAL1 localization. (**B**) The TSS1 region amplified using primer pair 5 was cloned into pGL3 basic vector and transfected into HeLa cells. Luciferase activity was measured after 36 hours and was normalized to Renilla activity. (**C**) SMARCAL1, RNAPII, H3K9Ac, and H3K9(Me)2 occupancy on *brg1* promoter was analyzed using primer pair 2 after treating HeLa cells with 2 μM doxorubicin for 10 min. The difference was significant for all with p-values  < 0.05. (**D**) SMARCAL1, RNAPII, H3K9Ac, and H3K9(Me)2 occupancy on *brg1* promoter was analyzed using primer pair 5 after treating HeLa cells with 2 μM doxorubicin for 10 min. The difference was significant for RNAPII, SMARCAL1 and H3K9(Me)2 with p values  < 0.05. GAPDH was used as the internal control in all these experiments. (**E**) Model explaining the regulation of *brg1* gene in the absence and presence of doxorubicin by SMARCAL1.

We next addressed how BRG1 was regulated. Previously we had observed that *brg1* expression was downregulated in *SMARCAL1* downregulated cells^24^. Was it possible that SMARCAL1 was regulating *brg1* transcription? To test our hypothesis, we used ChIP assay to probe the occupancy of SMARCAL1 on *brg1* promoter. Bioinformatic analysis using DBTSS (http://dbtss.hgc.jp) identified a putative transcription start site (TSS1) on the *brg1* promoter and we confirmed it using luciferase reporter assay (Fig. 4A,B). On the *brg1* promoter SMARCAL1 and RNAPII were found to co-localize approximately 800 bp upstream of TSS1 (Supplementary Fig. S8). Further, we found that SMARCAL1 was also localized at the putative transcription start site (Supplementary Fig. S8).

Finally, the occupancy of SMARCAL1 on *brg1* promoter was examined in cells after treatment with 2 μM doxorubicin for 10 minutes. We found that the occupancy of SMARCAL1 on the region amplified by primer pair 2 increased while, surprisingly, at this region the RNAP II and H3K9Ac occupancy decreased (Fig. 4C–E). We also analyzed the occupancy of RNAPII and SMARCAL1 at the putative transcription start site (pair 5 region) and found that the occupancy of both these proteins increased on treatment with doxorubicin (Fig. 4C–E).

### ADAAD, the bovine homolog of SMARCAL1, binds to *brg1* promoter and uses it as an effector for ATP hydrolysis

**Figure 5:**
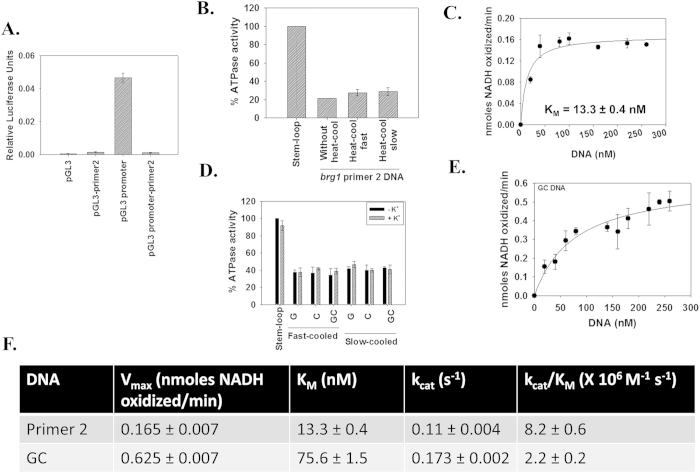
ADAAD can hydrolyze ATP using primer 2 as effector DNA. (**A**) The primer 2 region of *brg1* promoter functions as a repressor. The region was cloned into pGL3 basic vector as well as pGL3 promoter vector. The constructs were transiently transfected into HeLa cells and assayed for luciferase activity after 36 hours. In this experiment the luciferase activity was normalized with respect to Renilla. (**B**) ADAAD, the bovine homolog of SMARCAL1, hydrolyzes ATP in the presence of primer 2 DNA. 20 nM DNA was incubated with 0.1 μM ADAAD in the presence of ATP for 60 minutes at 37 °C. (**C**) The K_M_ for the interaction was calculated for ADAAD-primer 2 DNA interaction by measuring the ATPase activity with increasing DNA concentration. The reactions were incubated at 37 °C for 60 minutes. 0.1 μM ADAAD was used for this experiment. (**D**) ATPase activity induced by single-strand G, single-strand C, and double-strand GC DNA. 10 nM DNA was incubated with 0.24 μM ADAAD in the presence of ATP for 30 minutes at 37 °C. (**E**) The K_M_ for ADAAD-GC DNA was calculated by measuring the ATPase activity with increasing DNA concentration. The reactions were incubated at 37 °C for 30 minutes and 0.24 μM ADAAD was used in the reaction. (**F**) Comparison of kinetic parameters for the interaction of ADAAD with primer 2 DNA and GC DNA.

To understand the dynamics of *brg1* regulation, the region encompassed by primer pair 2 (henceforth, referred as primer 2 DNA) was amplified and cloned into pGL3 basic as well as promoter vector. We found that the primer 2 DNA of *brg1* promoter does not possess any promoter activity and that it functions as a repressor (Fig. 5A).

Previously we have shown that SMARCAL1 regulates *c-myc* expression by altering the conformation of the promoter region^24^. Therefore, we asked whether the protein can do the same in the case of *brg1* promoter. In a manner similar to the *c-myc* promoter^24^, the QGRS^25^ software (http://bioinformatics.ramapo.edu/QGRS/analyze.php) once again predicted a possible G-quadruplex comprising of G2-L2-G2-L4-G2-L2-G2 where G is guanine and L is loop comprised of any nucleotide on the reverse strand (Supplementary Table S2). Further, Mfold^26^ predicted that the DNA encompassing the G-quadruplex, termed as G DNA, can fold into a stem-loop structure (Supplementary Table S3).

We have used Active DNA-dependent ATPase A Domain (ADAAD), the homolog of SMARCAL1, for the biochemical and biophysical experiments as it is easier to purify and has been well-characterized with respect to its biochemical properties^27^,^28^.

The primer 2 DNA was found to function as an effector of ADAAD both in native form and when it was heat-cooled (Fig. 5B). The apparent K_M_ for ADAAD-primer 2 interaction was calculated to be 13.3 ± 0.5 nM indicating that primer 2 DNA was approximately 6-fold weaker effector than the stem-loop DNA (Fig. 5C). This interaction is also approximately 4-fold weaker than that between ADAAD and c*-myc* promoter^24^.

### ADAAD binds to the putative G-quadruplex with 5-fold weaker affinity

Next, we synthesized a 40-nt oligonucleotide spanning the predicted G-quadruplex. This oligonucleotide, called G DNA, can make a double-stranded DNA with its complementary sequence called C DNA (Supplementary Table S3; Supplementary Fig. S9). It should be noted that while both G and C oligonucleotides can fold into a stem-loop structure, only the G DNA is predicted to form a G-quadruplex.

ATPase assays showed that ADAAD can hydrolyze ATP in the presence G, C, and GC DNA (Fig. 5D). Further, there was no difference between fast-cooled and slow-cooled DNA as well as absence and presence of K^+^ (Fig. 5D). The K_M_ for ADAAD-GC DNA interaction was found to be 75.6 ± 1.5 nM, which is 5-fold weaker than that for ADAAD-primer 2 DNA interaction (Fig. 5E,F). Further, the catalytic efficiency of the ADAAD-primer 2 DNA was greater than that for ADAAD-GC DNA interaction (Fig. 5F). From these results, we concluded that though ADAAD can recognize the GC fragment, it is not sufficient for the interaction within the primer 2 DNA context.

### SMARCAL1 regulates *brg1* expression by altering the conformation of the promoter

**Figure 6:**
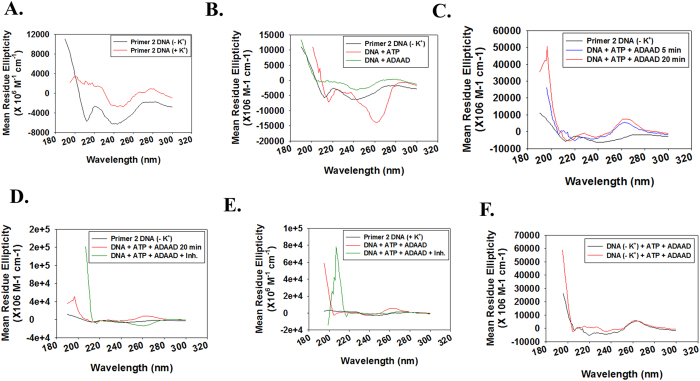
ADAAD alters the conformation of primer 2 DNA. (**A**) CD spectra for DNA alone in the absence and presence of K^+^. (**B**) CD spectra for primer 2 DNA (heat-cooled in the absence of K^+^) in the presence of ATP alone and ADAAD alone. (**C**) CD spectra for DNA alone and DNA incubated with 0.1 μM ADAAD and 0.5 mM ATP for 5 minutes at 37 °C. (**D**) Comparison of CD spectra of DNA alone, DNA with ADAAD and ATP incubated for 20 minutes at 37 °C before and after addition of 5 μM ADAADiN. ADAADiN was added after the DNA was incubated for 20 minutes in the presence of 0.1 μM ADAAD and 0.5 mM ATP at 37 °C. After addition, the CD spectra were recorded at 37 °C. As each scan takes 1 minute, and 5 scans were taken, the reaction with ADAADiN had been effectively incubated for 5 minutes. (**E**) CD spectra of DNA (heat-cooled in the presence of 100 mM K^+^) alone, DNA with protein and ATP incubated at 37 °C for 5 minutes, and after addition of 5 μM ADAADiN. (**F**) Comparison of CD spectra of DNA (heat-cooled in the absence and presence of 100 mM K^+^). In these reactions, 0.15 nM DNA, 0.1 μM ADAAD, 0.5 mM ATP and 10 mM MgCl_2_ was used. ADAADiN is indicated as Inh. within the figure. In both cases, DNA was heated to 94 °C for 3 minutes and slowly cooled to room temperature either in the absence or presence of 100 mM K^+^.

Next, we probed the conformational change in the primer 2 DNA using circular dichroism^29^. The primer 2 DNA alone in the absence and presence of K^+^, forms a positive peak at 280 nm (Fig. 6A). When primer 2 DNA (in the absence of K^+^) was incubated with ATP alone a negative peak at 260 nm was observed (Fig. 6B). Addition of ADAAD and ATP induced a conformational change in the primer 2 DNA resulting in a positive peak at 260 nm, which did not change with further incubation at 37 °C (Fig. 6C). The conformational change in the promoter region requires continuous ATP hydrolysis as addition of ADAADiN^19^, the inhibitor of ADAAD, resulted in disruption of this structure (Fig. 6D). Interestingly, addition of ADAADiN resulted in the formation of the negative 260 nm peak (Fig. 6D).

We also analyzed the conformational change induced in the primer 2 DNA that was heat-cooled in the presence of K^+^ and found that the DNA formed a positive peak at 260 nm peak in the presence of ATP and ADAAD, similar to the one formed in the absence of K^+^ (Fig. 6E,F).

From these experiments we concluded that binding of SMARCAL1 to the primer 2 region of *brg1* promoter results in a conformational change in the DNA. We hypothesize that RNAPII was possibly poised upstream of the transcription start site at region encompassed by primer 2 DNA in normal HeLa cells. Upon treatment with doxorubicin, SMARCAL1 induces a structural change in the DNA that releases RNAPII from its poised state. Simultaneously, there is increased binding of RNAPII at TSS1 resulting in increased transcription.

### Presence of both SMARCAL1 and BRG1 is necessary for the mutual upregulation and is specific to doxorubicin treatment

Next we wanted to understand whether the presence of both the protein was necessary for the mutual upregulation. Therefore, we compared the expression of SMARCAL1 and BRG1 in HeLa, HepG2, and A549 cells in the absence and presence of doxorubicin treatment. HeLa expresses both SMARCAL1 and BRG1 while A549 expresses only SMARCAL1 (Fig. 2C and Supplementary Fig. S10). In case of HepG2, BRG1 was detectable both at protein and transcript level but SMARCAL1 transcript was undetectable though we could observe small amount of protein (Supplementary Fig. S10). Further, we detected a doublet for BRG1 in HepG2 cells. Doxorubicin treatment did not result in upregulation of SMARCAL1 and BRG1 in A549. In HepG2 cells SMARCAL1 was not upregulated after doxorubicin treatment; however, in case of BRG1, the lower band (indicated as band 2) did not change while the upper band (indicated as band 1) showed upregulation. It has been proposed that BRG1 is phosphorylated during DNA damage repair^30^. We propose that the upper band of the doublet is possibly the phosphorylated form, while the lower band is the non-phosphorylated form, and therefore, it is the phosphorylated form that is changing as a response to doxorubicin treatment. However, we need additional experiments using antibodies recognizing the phosphorylated form to confirm our hypothesis. Based on the fact that the transcript levels of BRG1 and SMARCAL1 do not alter in A549 and HepG2 cells, we conclude that the expression of both the proteins is essential for the mutual upregulation (Supplementary Fig. S10).

To understand whether treatment with other DNA damaging agents would also result in the upregulation of BRG1 and SMARCAL1, we treated cells with 100 μM H_2_O_2_, a reagent known to induce base lesions as well as breaks in DNA, for 10 minutes^31^. As shown in Supplementary Fig. S4, this treatment results in DNA damage. However, we did not observe any change in the expression of BRG1 and SMARCAL1 indicating that this regulation was operative only when cells were treated with doxorubicin (Supplementary Fig. S10).

## Discussion

Gene expression in a cell is regulated by transcriptional networks. Transcription factors respond to extracellular signal and orchestrate the cellular response by activating or repressing gene expression^32^. As the DNA within a eukaryotic cell is packaged into chromatin it is imperative that the transcription factors work in conjunction with chromatin remodeling factors to orchestrate the response to biological signals.

Transcriptional networks, which consist of small recurring motifs, were first identified in prokaryotes and subsequently, in *S. cerevisiae*^33^,^34^,^35^. The simplest motifs are the feedback loops which can either be positive or negative^36^.

In this study, we have identified one such regulatory loop consisting of two ATP-dependent chromatin remodeling factors: BRG1 and SMARCAL1.

The occupancy of SMARCAL1 on the *brg1* promoter was a surprise finding. Bioinformatic analysis of region on c*-myc* and *brg1* promoter bound by SMARCAL1 showed that both these DNA molecules possess ability to form G-quadruplex as well as stem-loop structures. Thus, it is possible that SMARCAL1 regulates genes whose promoter possesses these characteristics. However, SMARCAL1 does not induce the formation of G-quadruplex on either of these promoters.

Biochemical assays showed that ADAAD, the bovine homolog of SMARCAL, can use this promoter as an effector to hydrolyze ATP and harness the energy released by hydrolysis to induce a conformational change in the structure. It should be noted that the K_M_ for the interaction with *brg1* promoter region is 4-fold weaker than the interaction with the *c-myc* promoter; thus despite structural similarities, SMARCAL1 can differentiate between the promoters.

The conformational change in the *brg1* promoter appears to correlate with transcription activation. We hypothesize that on the *brg1* promoter RNAPII is poised at primer 2 DNA. In the presence of appropriate signal, SMARCAL1 binds to the promoter at the primer 2 DNA creating a structure which results in removal of RNAPII from this site and thus relieving the transcription block. It is also possible that like its bacterial counterpart, HepA (also known as Rap A) protein, which is a component of prokaryotic RNA polymerase and regulates its function by facilitating its recycling during transcription, SMARCAL1 too modulates the activity of RNA pol II^37^. Further experiments are needed to delineate the mechanism of transcription activation of *brg1* by SMARCAL1.

Our experimental data also shows that BRG1 is present on *SMARCAL1* promoter and the occupancy of this protein increases when DNA is damaged, indicating that BRG1 also positively regulates *SMARCAL1*, thus, creating a regulatory loop.

The presence of this regulatory motif led us to postulate that BRG1 and SMARCAL1 must be required for the same process such that the mutual upregulation between these two proteins can amplify the response^36^. It is well-documented that both BRG1 and SMARCAL1 participate in repairing DNA damage induced by IR^17^,^18^,^21^,^22^,^38^. The presence of two different ATP-dependent chromatin remodeling factors at the site of repair at the same time might be necessitated by their ability to perform different functions despite possessing the conserved helicase motifs and belonging to the same protein family^2^. Earlier reports have suggested that the interaction of BRG1 with γH2AX results in enhanced histone H3 acetylation while SMARCAL1 is required for stabilizing replication forks during DNA damage^17^,^21^,^22^,^39^. Further, both BRG1 and SMARCAL1 have been shown to interact with γH2AX though neither of them is required for its recruitment at the site of DNA damage^18^,^22^,^39^,^40^. It is, thus, possible that these two proteins are regulating each other as they both mediate double-strand break repair.

The co-regulation reported in this paper does not appear to be operative under all conditions. For example, we have analyzed the levels of SMARCAL1 and BRG1 during differentiation of K562 in response to PMA and found that during this process the regulatory loop is not operative^24^.

We believe that the regulatory loop identified in HeLa cell line when it is treated with doxorubicin to induce DNA damage is one of the simplest network existing in the cell. We hypothesize that there are more complex networks involving ATP-dependent chromatin remodeling factors and histone modifying enzymes present in mammalian cells that are yet to be identified. These networks would then feed on to other transcription factors creating feed forward loops enabling the cell to respond to external stimuli.

## Methods

### Reagents

Dulbecco’s Modified Eagle’s Medium (DMEM), penicillin-streptomycin cocktail, amphotericin B, sodium bicarbonate, fetal bovine serum, TRIzol reagent, primers, Hoechst 33342, doxorubicin, thymidine and Escort^TM^ transfection reagent were purchased from Sigma-Aldrich (USA). Zeocin was obtained from Invitrogen (USA). Restriction enzymes, Turbofect, M-MuLV Reverse Transcriptase kit, Hi-fidelity PCR enzyme mix, FastAP thermo sensitive Alkaline Phosphatase and the INSTAclone TA-cloning kit were purchased either from MBI Fermentas (USA) or from NEB (USA). QIAquick gel extraction kit was purchased from Qiagen (USA). Protein A-CL agarose bead resin was purchased from Merck (India) and Protein-G fast flow bead resin was purchased from Merck-Millipore (USA). 2X SYBR Green PCR master mix, micro-amp Fast 96-well reaction plates (0.1 ml) and micro-amp optical adhesive films were purchased from Applied Biosystems (USA). 2X fast SYBR Green PCR master mix was purchased from Kapa Biosystems (USA). Dual luciferase assay kit was purchased from Promega (USA). For western blotting, Immobilon-P PVDF membrane was purchased from Merck-Millipore (USA). X-ray films, developer, and fixer were from Kodak (USA).

### Antibodies

The various primary antibodies, unless otherwise mentioned, were purchased from Sigma-Aldrich (USA), Cell Signalling Technology (USA) or Merck (India). In our experiments, BRG1 antibody (Sigma-Aldrich; Catalog #B8184), RNAPII (Cell Signalling Technology; Catalog 2629S), H3K9Ac (Sigma-Aldrich; Catalog #H0913); H3K9(Me)2 (Sigma-Aldrich; Catalog #D5567), γH2AX (Sigma-Aldrich; Catalog #H5912) and β-actin (Sigma-Aldrich; Catalog #A1978) were used. Anti-GFP antibody was purchased from Life Technologies (Catalog #A11120). Monoclonal antibodies against bovine SMARCAL1 were a kind gift from Dr. Joel W. Hockensmith, University of Virginia. The TRITC- and FITC-conjugated anti-rabbit (Merck; Catalog #RTC2) and anti-mouse (Merck; Catalog #FTC3) antibodies and the HRP-conjugated anti-mouse IgG (Merck; Catalog #HPO5) and anti-rabbit IgG (Merck; Catalog #HPO3) antibodies were obtained from Merck (India). Monoclonal anti-mouse Sp1 antibody (Millipore; Catalog #07-645) was a kind gift from Prof. B.N. Mallick, School of Life Sciences, JNU. Polyclonal antibody against SMARCAL1 was generated as explained previously^19^.

### Primers

The list of primers used in cloning, quantitative real-time RT-PCR and ChIP are given in Supplementary Tables S4–7. All oligonucleotides were synthesized by Sigma-Aldrich (USA).

### Vectors

The pCMV-SPORT6-SMARCAL1 vector was a kind gift from Dr. Joel W. Hockensmith, University of Virginia. The pcDNA3.1 (+) zeocin vector was a kind gift from Dr. Ranjana Arya, School of Biotechnology, JNU. pEGFP-C3 and pGL3 vectors were a kind gift from Prof. S.K. Goswami, School of Life Sciences, JNU.

### Bacterial Strains

The *E. coli* strain JM109 used for cloning was purchased from Merck, India.

### Construction of GFP-SMARCAL1 construct

The GFP tag was released along with its promoter from the pEGFP-C3 vector and cloned into the pcDNA 3.1 (+) zeocin vector between NdeI and BamHI sites creating pcDNA 3.1 (+) Zeo-GFP vector. For cloning SMARCAL1, pCMV-SPORT6-SMARCAL1 was used as the template and the gene was amplified using Hi-fidelity PCR enzyme with gene-specific primers. The amplified product was gel eluted and cloned into pTZ57R/T vector. The cloned insert was released using BamHI. pcDNA 3.1(+) Zeo-GFP vector was linearized with BamHI and given alkaline phosphatase treatment. Both insert and vector were then ligated to generate pcDNA 3.1(+) Zeo-GFP −SMARCAL1 construct.

### Construction of LAP-SMARCAL1 construct

The LAP tag cloning into the pcDNA 3.1(+) Zeo vector was a two-step process. Both pcDNA 3.1(+) Zeo and pcDNA5.0FRT/LAP-AURKB (a kind gift from Prof. Todd Stukenberg, University of Virginia) vectors were digested with BglII and ApaI to release the LAP-AURKB fragment with sticky ends compatible with the pcDNA 3.1(+) zeo backbone. The two fragments were ligated to form pcDNA3.1 (+) Zeo-LAP-AURKB construct. The AURKB fragment was later released using XhoI to create pcDNA 3.1(+) Zeo-LAP vector. SMARCAL1 gene was amplified using gene-specific primers and pCMV-SPORT6-SMARCAL1 as the template. The amplified product was gel eluted and cloned into pTZ57R/T vector. XhoI was used to release the cloned insert. pcDNA 3.1(+) Zeo-LAP vector was linearized with Xho I and was given alkaline phosphatase treatment. Both insert and vector were then ligated to generate pcDNA 3.1(+) Zeo-LAP-SMARCAL1 construct.

### Construction of pGL3-promoter construct

*SMARCAL1* promoter was amplified from HeLa genomic DNA using specific primers with Hi-fidelity PCR enzyme. The amplified product was cloned into pTZ57R/T vector and was confirmed by restriction digestion, followed by sequencing. *SMARCAL1* promoter region was then released from the T/A vector and cloned into appropriate pGL3 vector. The construct was confirmed using restriction digestion and used for further analysis. All the deletion constructs for *SMARCAL1* promoter were cloned by amplifying the sequences using specific primers.

In case of *brg1* promoter, the primer pair 2 and TSS1 region was amplified using the ChIP primer pairs 2 and 5 respectively and cloned into T/A vector. It was then released from the T/A vector and cloned into appropriate pGL3 vector.

### Cell culture and transfection

All cell lines were obtained from National Centre for Cell Science, Pune, India, and cultured in DMEM containing 10% fetal bovine serum and 1% penicillin-streptomycin-amphotericin B antibiotic cocktail at 37 °C in the presence of 5% CO_2_. Cells seeded to a confluency of 50–70% were transfected with various plasmid constructs using either Turbofect (Thermo Scientific) or Escort^TM^ transfection reagent (Sigma-Aldrich) according to the manufacturer’s protocol. In case of transient transfections, the cells were harvested and analyzed 36–48 hours post-transfection. For stable transfections with shRNA, cells were subjected to selection 36–48 hours post-transfection with the appropriate antibiotic at pre-standardized concentrations.

### Immunofluorescence

HeLa cells (2 × 10^5^ cells) were seeded on a coverslip in a 35 mm culture dish. After desired treatment, cells were processed by washing 3–4 times with 1X PBS followed by fixing with a 1:1 mixture of methanol and acetone for 10 min and subsequently, permeabilizing with 0.5% Triton X-100 in 1X PBS for 10 min. The cells were washed with 1X PBS and blocked in 2% BSA at 4 °C for 16 hours. After washing with 1X PBS, the cells were incubated with the desired cocktail of primary antibodies in 2% BSA at 37 °C for 30 minutes. The cells were then washed 3–4 times with 1X PBS and incubated with the mixture of either TRITC or FITC-conjugated secondary antibodies and Hoechst 33342 (1:1000) in 2% BSA at 37 °C for 30 minutes. The cells were then washed 3–4 times with 1X PBS, the coverslips were mounted on slides and viewed using a confocal microscope (Olympus) under a 60X oil immersion objective.

### Cell synchronization

HeLa cells were counted and seeded in culture dishes. For G1/S arrest, cells were synchronized by double thymidine block. Briefly, asynchronously growing cells were incubated with 2 mM thymidine for 18 hours, followed by washing with 1X PBS and releasing cells into fresh medium for 8 hours and then incubating again with 2 mM thymidine for another 16 hrs. The G1/S synchronized cells were then finally released into fresh media and used for various experiments.

### Cell extract preparation

Cell extracts were made using either RIPA lysis buffer (50 mM Tris-Cl pH 7.5, 300 mM NaCl, 2 mM EDTA, 1% v/v NP-40, 0.5% w/v sodium deoxycholate, 1% w/v sodium dodecyl sulphate) or urea lysis buffer (90% 8.8 M urea, 2% 5 M NaH_2_PO_4_ and 8% 1 M Tris-Cl pH 8.0). Briefly, cells were grown in 100 mm culture dishes to a confluency of 75–80%, harvested and thoroughly washed thrice with PBS. The cells were pelleted at 2500 rpm for 10 minutes at 4 °C and then resuspended in the appropriate lysis buffer. The cells were incubated on ice for 15 minutes with regular mild tapping followed by sonication (5 cycles of 30 sec on/off). The sonicated cell suspension was centrifuged at 13000 rpm for 10 minutes at 4 °C. The supernatant was collected and used for further experiments. Cell extract preparation using urea lysis buffer was done according to protocol described by Reisman *et al*.^41^. The protein concentration was determined using Bradford reagent.

### Western blotting

100 μg of the cell extract was electrophoresed on 9% SDS gel and transferred onto PVDF membrane by electro-blotting at 30 V for 16 hours at 4 °C. The blots were blocked in 5% (w/v) BSA in PBS (2.25 mM NaH_2_PO_4_, 145.5 mM NaCl, pH 7.2) for 2 hours at room temperature. The membrane was incubated with primary antibody for 1 hr. The membrane was then washed for 10 min three times in 1X PBST (0.05% Tween-20 in PBS) and incubated in secondary antibody for 45 minutes. After secondary incubation the blots were washed again with 1X PBST rinsed with 1X PBS and finally developed using the Enhanced Chemiluminiscence method (Sigma-Aldrich).

### Co-Immunoprecipitation

The appropriate antibody was incubated with HeLa cell extract made in RIPA lysis buffer at 4 °C for 16 hours. Pre-equilibrated protein A-agarose beads (30 μl) were then added and incubated at 4 °C for additional 4 hours. The immunoprecipitated proteins were then analyzed by western blot.

### RNA isolation and cDNA preparation

Total RNA was extracted using the TRIzol as per the manufacturer’s protocol. RNA concentrations were determined using NanoDrop 2000 (Thermo Fisher Scientific, USA) and equal amount of RNA from various samples was used for preparing cDNA using random hexamer primers according to the manufacturer’s protocol.

### Quantitative real-time RT-PCR

Quantitative real-time RT-PCR was performed with 7500 Fast Real-Time PCR system (ABI Biosystems, USA) using gene-specific primers designed either for exon-exon junctions (transcript estimation) or to specific promoter sequences (for ChIP analysis). For each reaction of 15 μl, samples were prepared in triplicates and the data obtained was analyzed using Fast 7500 software provided by manufacturer. The p-value was calculated using GraphPad prism or Sigma-Plot.

### Chromatin Immunoprecipitation (ChIP)

ChIP was performed according to the X-ChIP protocol provided online by Abcam (http://www.abcam.com/ps/pdf/protocols/x_CHip_protocol.pdf). The bound DNA was eluted using fresh elution buffer (1% sodium dodecyl sulphate and 100 mM NaHCO_3_). The eluted DNA was purified using phenol-chloroform and precipitated as mentioned in protocol. The resuspended DNA was used for ChIP-PCR using standardized primers.

### Dual-luciferase reporter assay

Cells were co-transfected, with pGL3- promoter constructs or pGL3-empty vector and pRL-TK, using turbofect. The luciferase assay was performed 36 hours after transfection using the Dual-luciferase reporter assay kit (Promega) and the luciferase activity was measured and normalized with respect to the controls.

### Purification of ADAAD and ATPase assays

ADAAD was purified and ATPase assays were done as explained in Nongkhlaw *et al*.^28^.

### CD spectra

CD spectra were recorded using Chirascan (Applied Photophysics). Briefly, CD spectra of 160 nM DNA were recorded in 1 mM sodium phosphate buffer (pH 7.0) in the presence of 0.5 mM ATP, 10 mM Mg^+2^, 100 mM K^+^ and 0.1 μM ADAAD. Spectra of appropriate buffer conditions were also taken at each time point. The spectra reading for each condition was subtracted from the appropriate buffer reading and plotted as a function of the wavelength.

For ATPase assays as well as for CD spectra, primer pair 2 region was amplified using appropriate primers. The amplicon was agarose gel-purified and used in these experiments. DNA fragments used for the assays were either used directly without any heat/cool treatment or were heated at 95 °C for 3 minutes followed by slow/fast cooling. For assays requiring K^+^, 1M KCl was added to the purified DNA to a final concentration of 100 mM.

### FACS analysis

For FACS, HeLa cells were synchronized using double thymidine block. After release, the cells at various time points were harvested by trypsinization. The collected cells were then washed with 1X PBS 2–3 times. Subsequently, the cell pellet was fixed with chilled 70% ethanol by gently adding it from the side of the tube along with vortexing. The fixed cells were stored at 4 °C for 2–3 hours. Just before analysis, ethanol was removed and the cells were resuspended in 500 μl PBS supplemented with 0.5 μg/ml of RNase and16 μg/ml propidium iodide. The cells were incubated at 37 °C in dark for 1 hour and used directly for sample run using BD Biosciences FACS Calibur flow cytometer. Post data acquisition, cell-cycle analysis was done using ModFit software.

### Comet assay

Single cell gel electrophoresis was performed as described by Nandhakumar *et al*.^42^. Briefly, HeLa cells after appropriate treatment were washed in cold 1X PBS, trypsinized and resuspended in ice cold PBS. The cell suspension containing 10^4^cells/slidewas embedded in 100 μl of 1% low-melting agarose (Sigma-Aldrich, USA) in 1X PBS and spread onto microscopy slides coated with 1% normal-melting agarose. The cells were lysed in the lysis solution (2.5 M NaCl, 100 mM Na_2_EDTA, 10 mM Tris base, 8 g/L NaOH to pH 10; 1% Triton X-100, and 10% DMSO) at 4 °C for 2 hours. Following lysis, the slides were placed for 20 min in a tank with cold electrophoresis buffer (300 mM NaOH, 1 mM Na_2_EDTA, HCl to pH 12.5) and electrophoresed for 30 min at 25 V and 300 mA. The slides were then neutralized with 0.4 M Tris-Cl pH 7.5 and the DNA was stained with ethidium bromide (0.5 mg/ml; Sigma-Aldrich). The slides were analyzed using fluorescence microscope (Nikon) at 10X magnification.

## Additional Information

**How to cite this article**: Haokip, D. T. *et al*. Transcriptional Regulation of Atp-Dependent Chromatin Remodeling Factors: Smarcal1 and Brg1 Mutually Co-Regulate Each Other. *Sci. Rep.* **6**, 20532; doi: 10.1038/srep20532 (2016).

## Supplementary Material

Supplementary Information
