# Supplementary material for: Transcriptional Regulation of Atp-Dependent Chromatin Remodeling Factors: Smarcal1 and Brg1 Mutually Co-Regulate Each Other
Source: Sci Rep. 2016 Feb 4;6:20532. doi: 10.1038/srep20532 (PMC4740806; doi:10.1038/srep20532)
Supplement: Supplementary Information [file srep20532-s1.doc]

**SUPPLEMENTARY INFORMATION**

**TRANSCRIPTIONAL REGULATION OF ATP-DEPENDENT CHROMATIN REMODELING FACTORS: SMARCAL1 AND BRG1 MUTUALLY CO-REGULATE EACH OTHER.**

Dominic Thangminlen Haokip^1^, Isha Goel^1^, Vijendra Arya, Tapan Sharma, Reshma Kumari^1^ Rashmi Priya, Manpreet Singh^2^, and Rohini Muthuswami**^§^**

Chromatin Remodeling Laboratory, School of Life Sciences, Jawaharlal Nehru University, New Delhi 110067

**^§^Corresponding Author:** Rohini Muthuswami, School of Life Sciences, Jawaharlal Nehru University**,** New Delhi, India

**Email:** [rohini_m@mail.jnu.ac.in](mailto:rohini_m@mail.jnu.ac.in)

^1^ **Equal First Authors:** Dominic Thangminlen Haokip and Isha Goel contributed equally to this paper.

^2^**Current Address:** Thapar University, Patiala, Punjab, India.


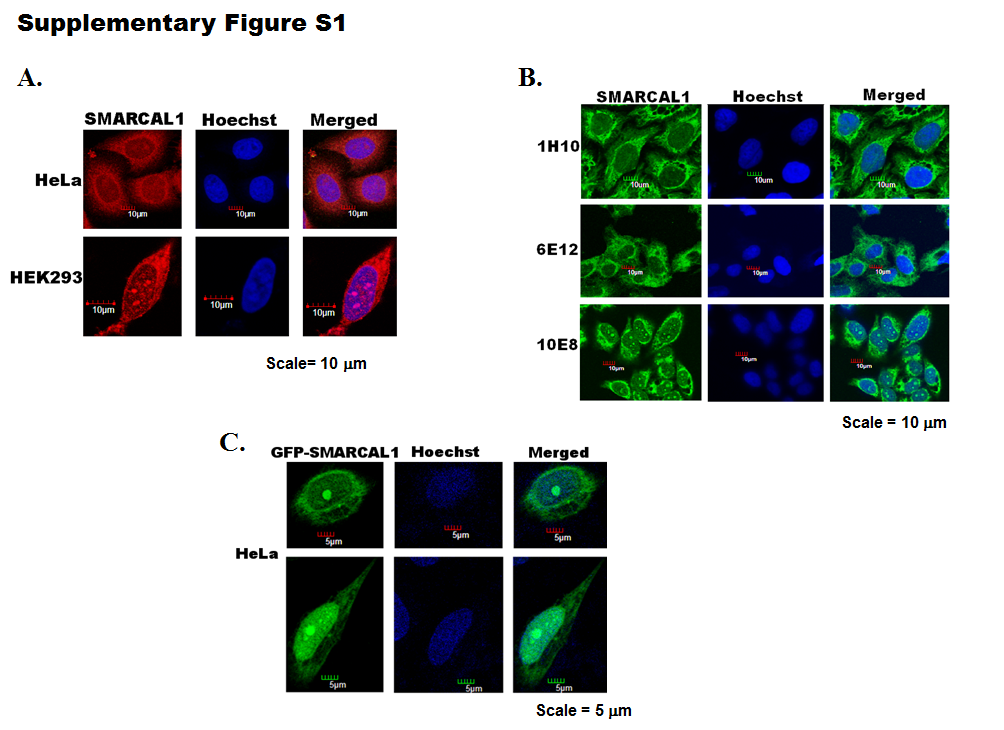


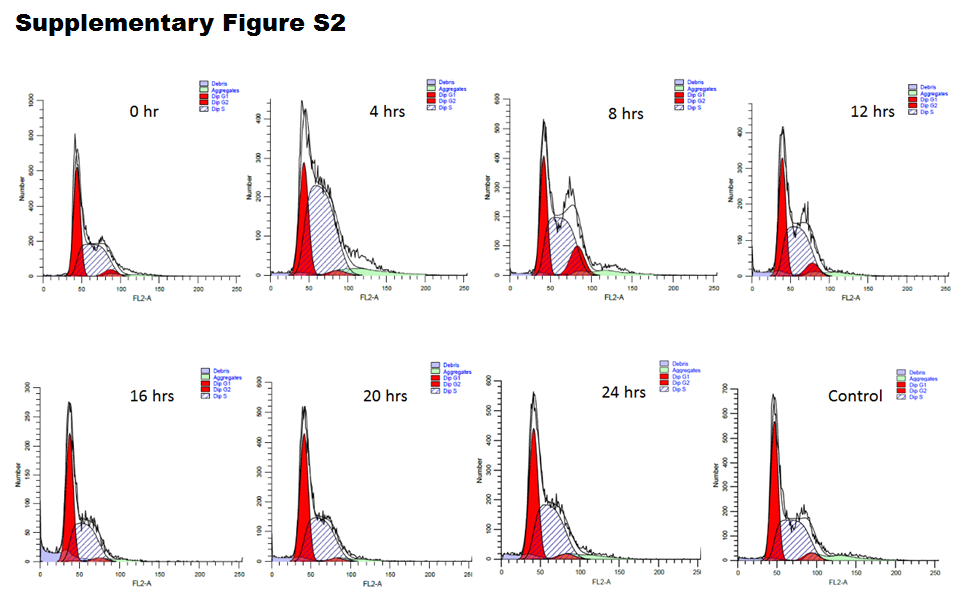


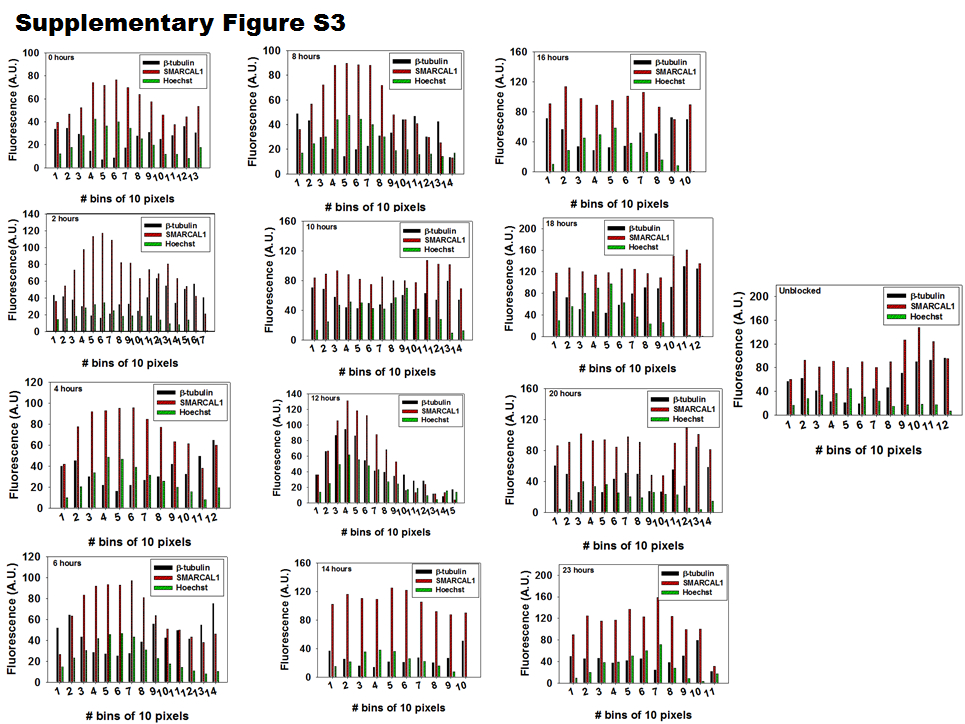


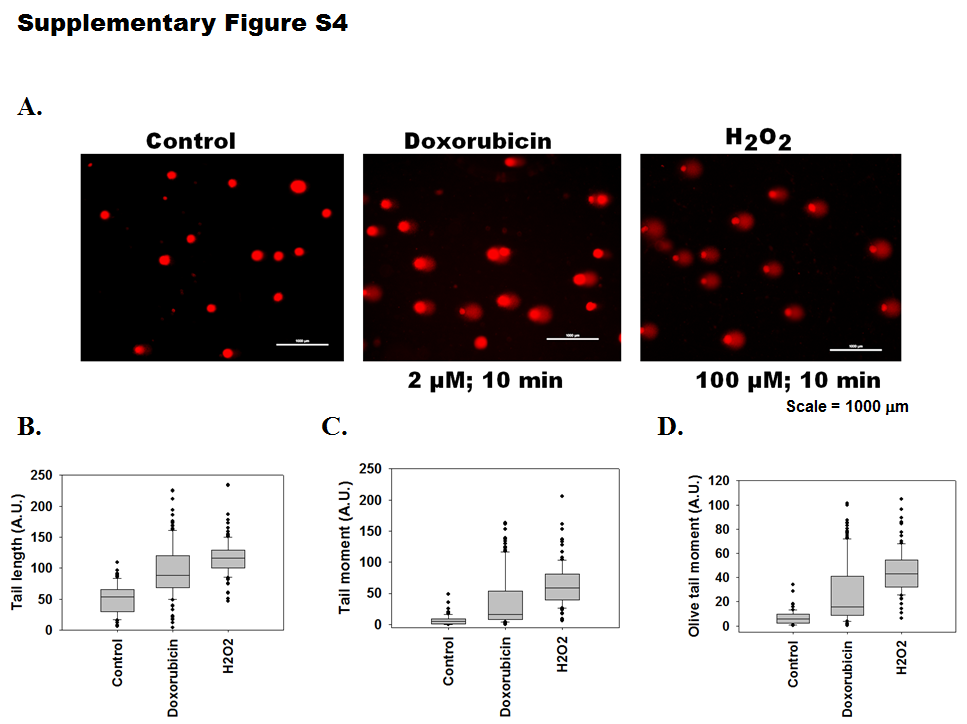


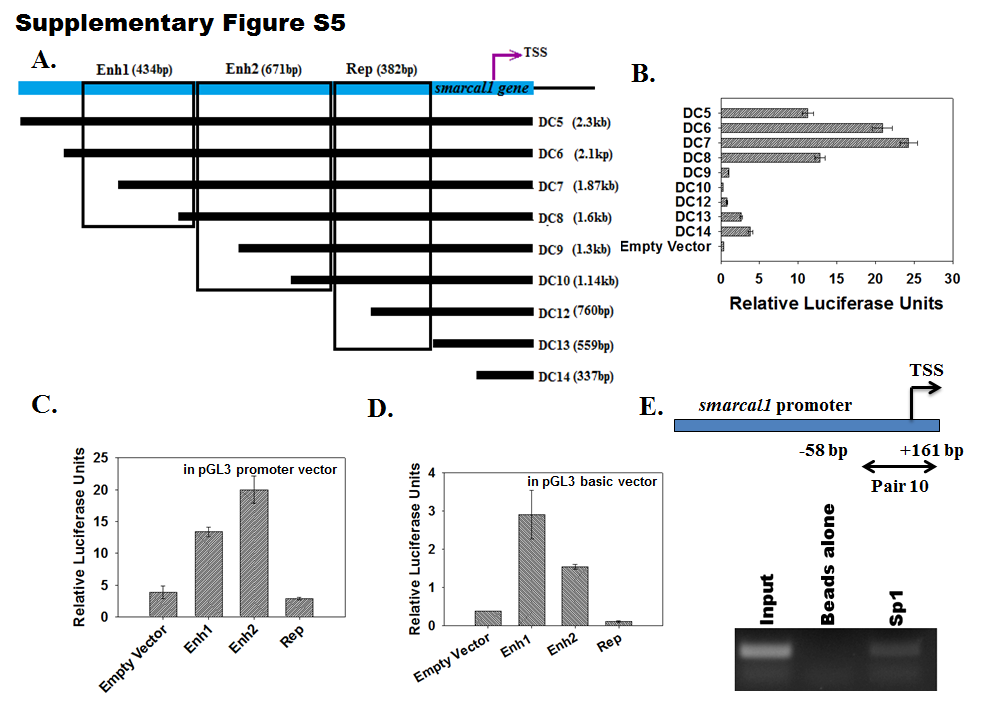


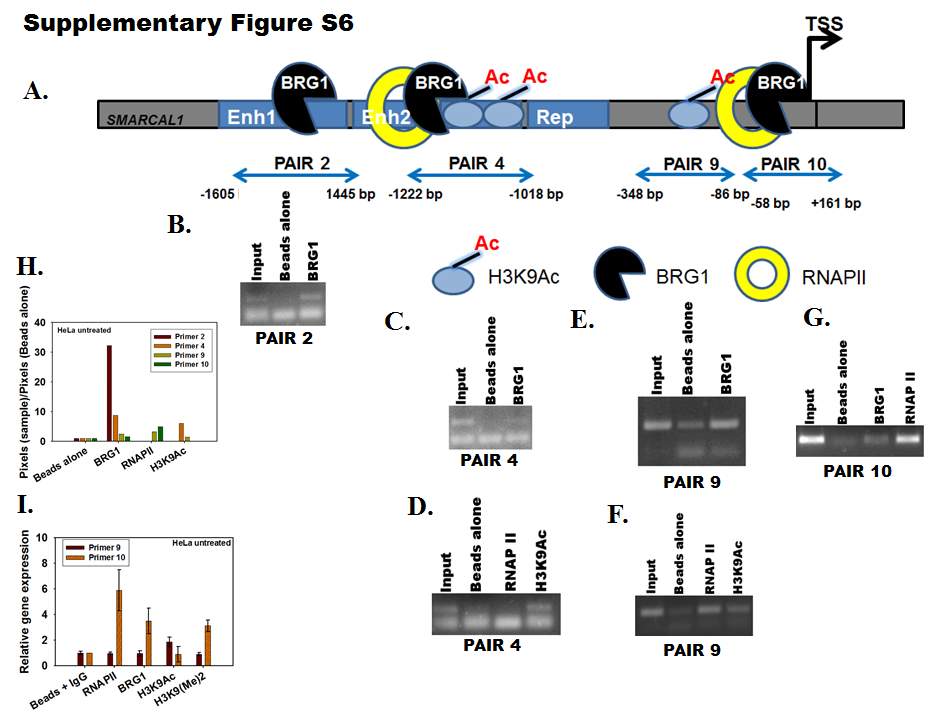


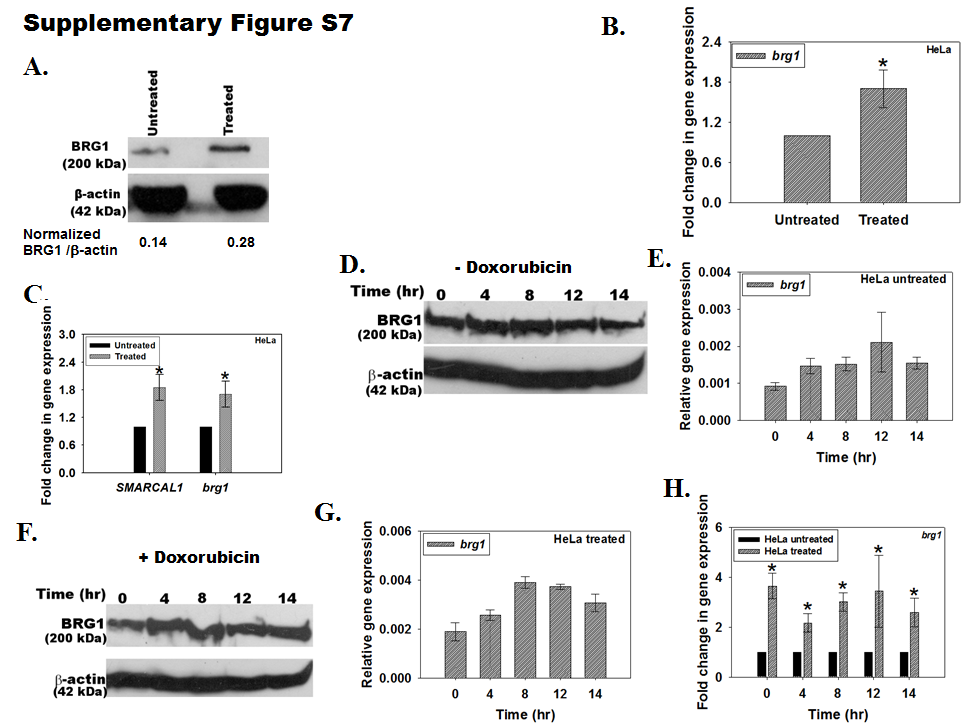


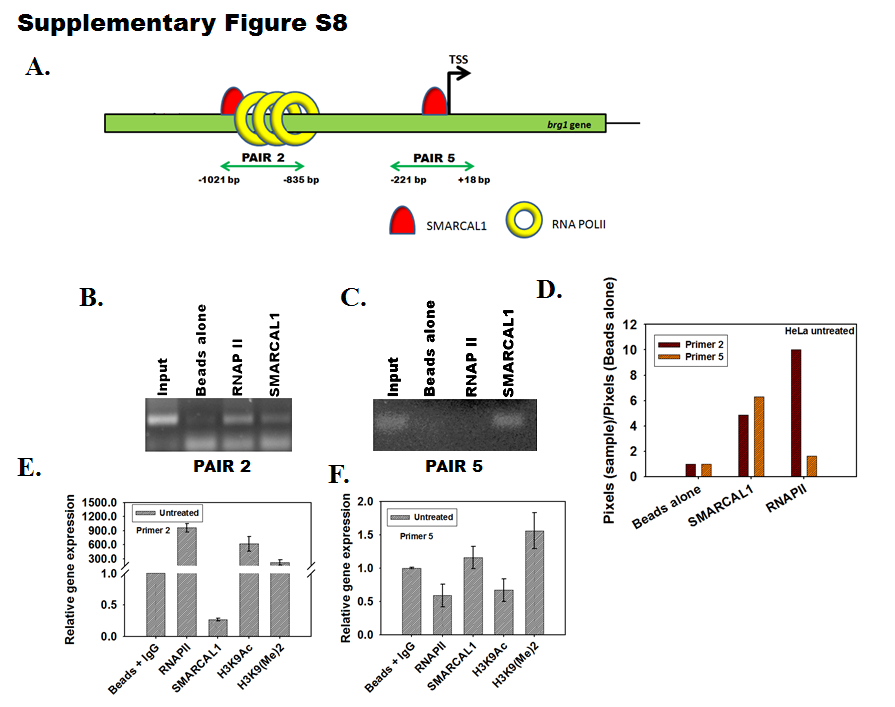


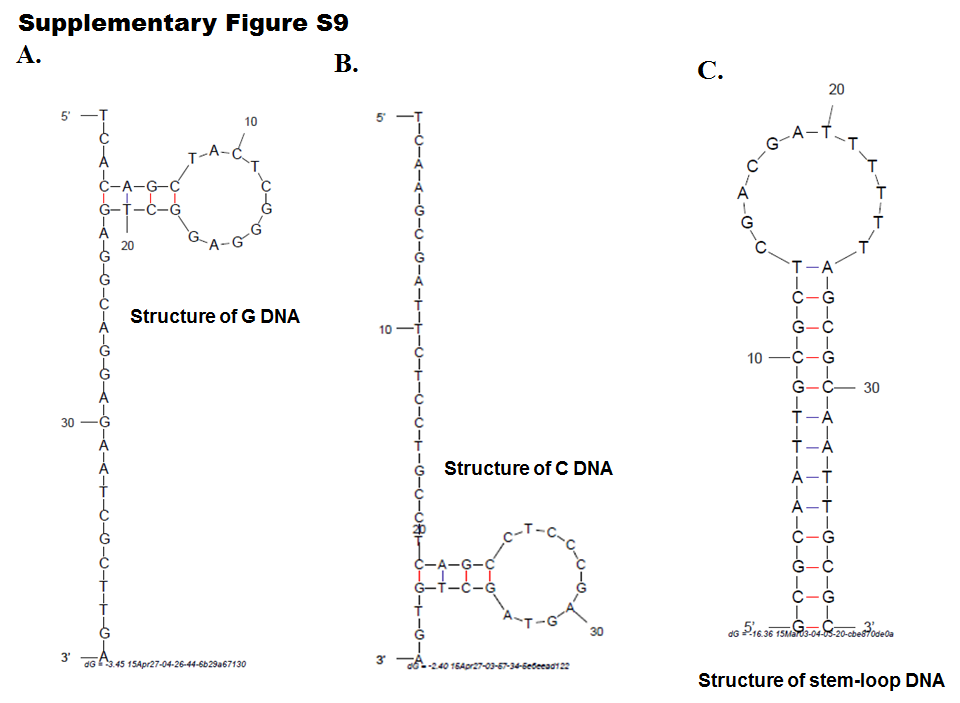


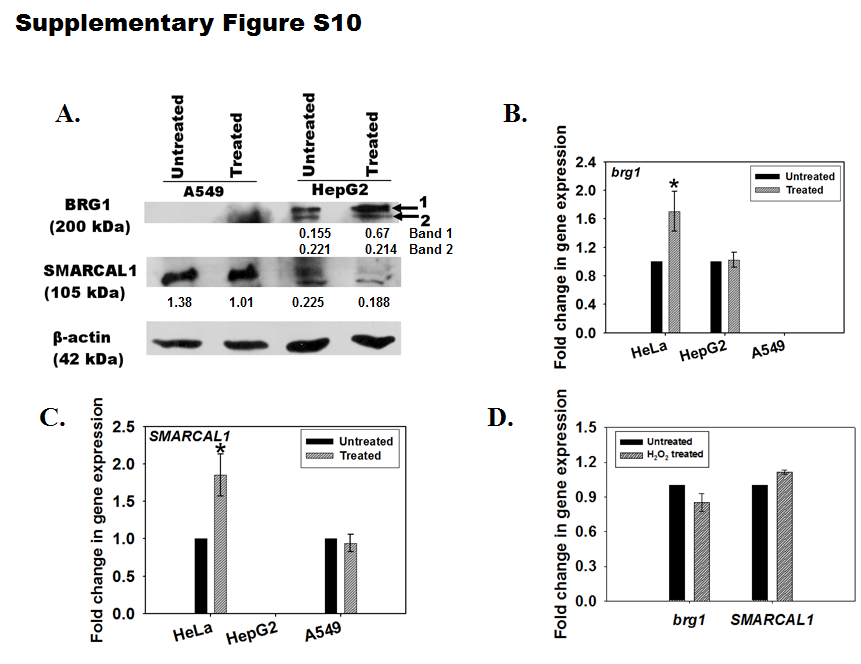


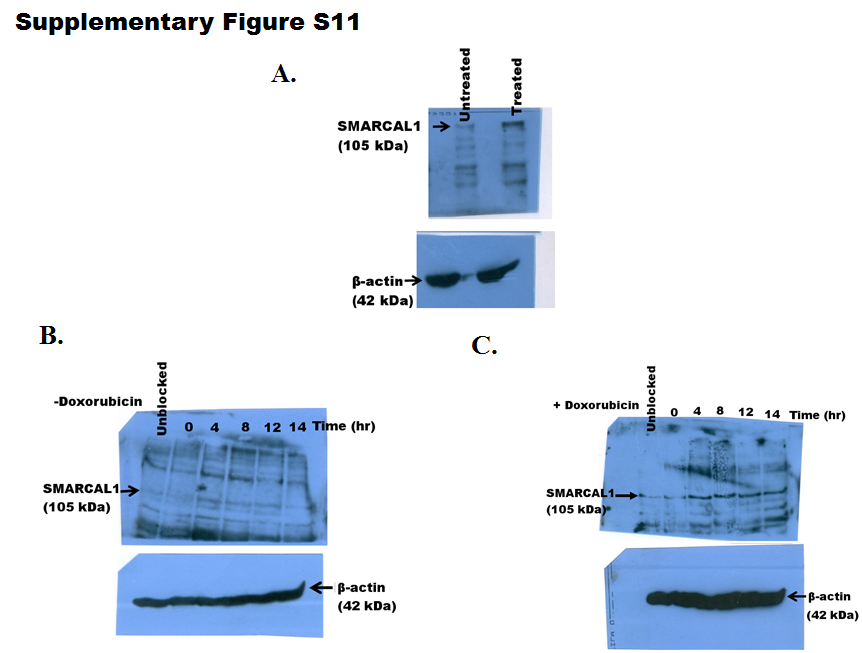


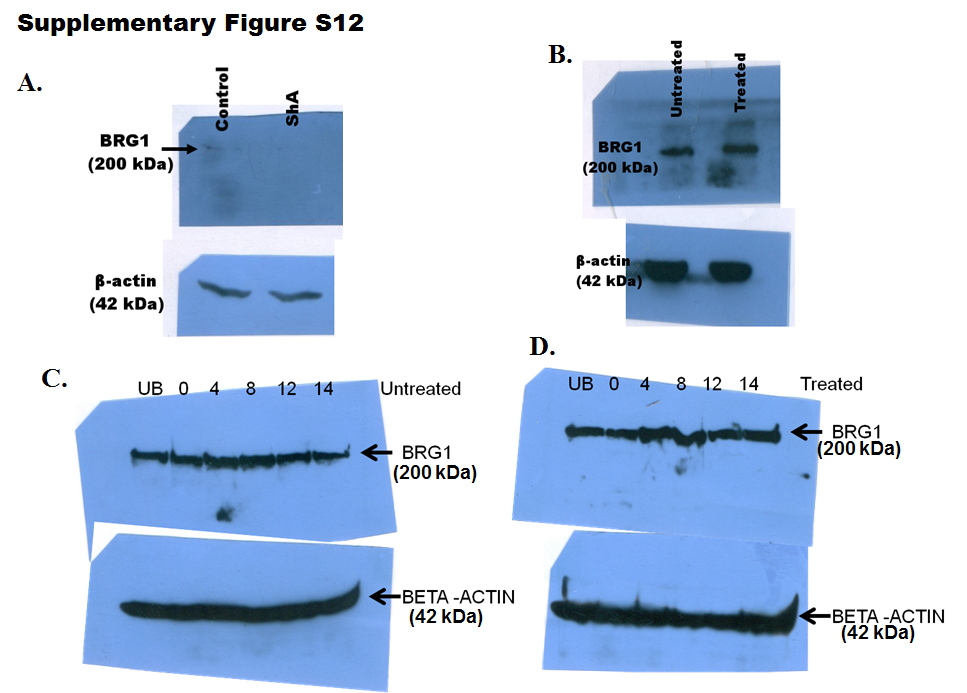


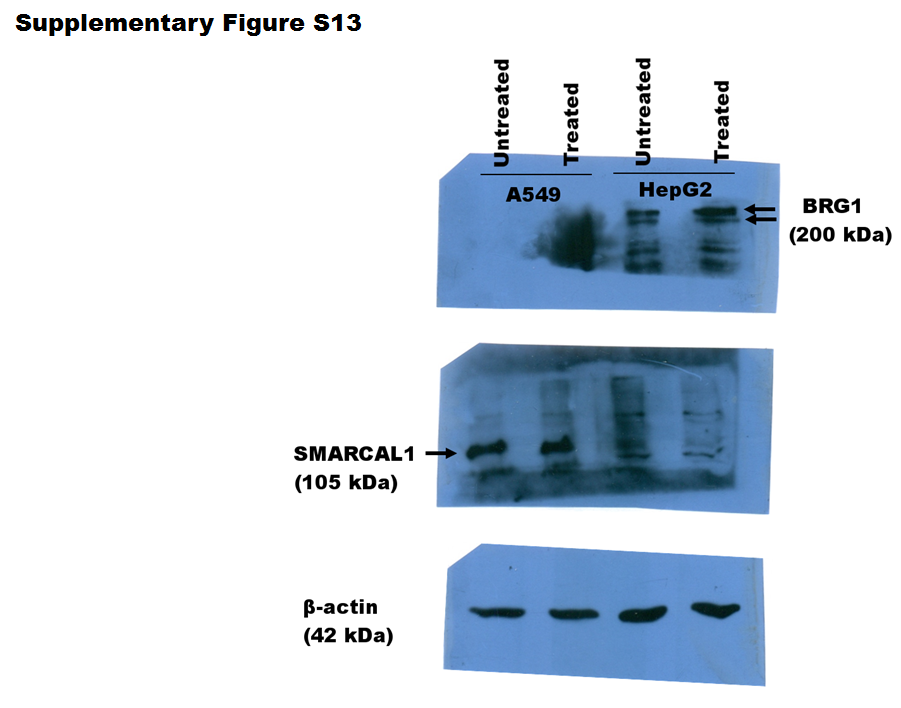


**Supplementary Table S1.** FACS analysis of HeLa cells after release from double thymidine block.

| **Time-point** | **G1 (%)** | **S (%)** | **G2/M (%)** | **Total events scored** | **Debris/Aggregates**  **events** |
| --- | --- | --- | --- | --- | --- |
| 0 h | 44.91 | 50.77 | 4.33 | 34335 | 3237 |
| 4 h | 27.79 | 69.21 | 2.99 | 32956 | 3506 |
| 8 h | 30.10 | 57.5 | 12.41 | 32097 | 11101 |
| 12 h | 37.14 | 54.39 | 8.47 | 11564 | 1633 |
| 16 h | 46.81 | 48.03 | 5.17 | 21969 | 2999 |
| 20 h | 46.81 | 48.74 | 4.46 | 24371 | 3129 |
| 24 h | 42.79 | 53.39 | 3.83 | 33103 | 4407 |

**Supplementary Table S2.**

The sequence of primer 2 DNA of *brg1* promoter where SMARCAL1 occupancy was found:

| Forward sequence 5′ 🡪3′ | Reverse sequence 5′ 🡪3′ |
| --- | --- |
| CCCAGGCTGGAGTGCAATAGTACGATCTCGGCTCACTACAATCTCCACCTCCTGGGCTCAAGCGATTCTCCTGCCTCAGCCTCCCGAGTAGCTGTGACTACAGGCGTGTGCCACCACGCCCGGCTAATTTTTTGTATTTTTAGTAAAGACGGGGTTTCACCGTGTTAGCCAGGATAGTCTCATCTCC | GGAGATGAGACTATCCTGGCTAACACGGTGAAACCCCGTCTTTACTAAAAATACAAAAAATTAGCCGGGCGTGGTGGCACACGCCTGTAGTCACAGCTACTC***GG*GA*GG*CTGA*GG*CA*GG***AGAATCGCTTGAGCCCAGGAGGTGGAGATTGTAGTGAGCCGAGATCGTACTATTGCACTCCAGCCTGGG |

The sequence highlighted in bold and underlined was predicted by QGRS^1^ mapper to form G-quadruplex.

**Supplementary Table S3. Sequence of G, C, and stem-loop oligonucleotides used in our study. The putative G-quadruplex forming sequence is in bold.**

| **Oligonucleotide** | **Sequence (**5′🡪3′) | **Nucleotide length (nt)** | **Formation of G-quadruplex**^1^ | **Formation of stem-loop structure**^2^ |
| --- | --- | --- | --- | --- |
| G | TCACAGCTACTC***GGGAGGCTGAGGCAGG***AGAATCGCTTGA | 34 | Yes; score 19 | Yes; ΔG = -3.45 kcal/mol |
| C | TCAAGCGATTCTCCTGCCTCAGCCTCCCGAGTAGCTGTGA | 34 | No | Yes; ΔG = -2.40 kcal/mol |
| Stem-loop | GCGCAATTGCGCTCGACGATTTTTTAGCGCAATTGCGC | 38 | No | Yes; ΔG = -16.36 kcal/mol |

G and C are complementary to each other and can form double-stranded DNA.

**Supplementary Table S4**. List of primers used for cloning *smarcal1* promoter along with the amplicon size.

| Amplicon size (bp)  (Location with respect to TSS) | Annealing temperature (^o^C) | Forward primer (5'→3') | Reverse primer (5'→3') |
| --- | --- | --- | --- |
| DC5 (2353bp)  (-2237to +115) | 63 | **GGTACC**GTGTATTTCATGCCACTCTGC | **AGATCT**ATGGCGTGACACTTGCTAACAT |
| DC6 (2127bp)  (-2012to +115) | 66 | **GGTACC**GCAACATTCATATGCACACTGATAGAG | **AGATCT**ATGGCGTGACACTTGCTAACAT |
| DC7 (1877bp)  (-1762to +115) | 60 | **GGTACC**GTAGTGCGGTTAAATGAGGTAAC | **AGATCT**ATGGCGTGACACTTGCTAACAT |
| DC8 (1642bp)  (-1527to +115) | 66 | **GGTACC**GGCTTTCAGGACCTAGTGTTTGGC | **AGATCT**ATGGCGTGACACTTGCTAACAT |
| DC9 (1356bp)  (-1229to +115) | 60 | **GTACC**TAACCGTCCACTCGGAAGAC | **AGATCT**ATGGCGTGACACTTGCTAACAT |
| DC10 (1142bp)  (-1027to +115) | 72 | **GGTACC**GATCGTAGGGGTGGGAGTGGGGCG | **AGATCT**ATGGCGTGACACTTGCTAACAT |
| DC12 (762bp)  (-647to +115) | 64 | **GGTACC**GATAGATACTTCGTGGTGGGGG | **AGATCT**ATGGCGTGACACTTGCTAACAT |
| DC13 (559bp)  (-444to +115) | 70 | **GGTACC**GGTGTGTCCTGCCCGCTGCTTC | **AGATCT**ATGGCGTGACACTTGCTAACAT |
| DC14 (337bp)  (-222to +115) | 60 | **GGTACC**GTGGTTATTGATGGCTGGTAAG | **AGATCT**ATGGCGTGACACTTGCTAACAT |
| Enh 1(434bp)  (-1896to -1436) | 63 | **GGTACC**TAATCTTCTCTCAAGAGGAGGGGC | **AGATCT**AAAGTGGTGCACCCTGGAAGTTG |
| Enh 2 (671bp)  (-1462-791) | 60 | **GGTACC**CGCCCCGCCCATGACGTTGCAGC | **AGATCT**CACCTCCAAACTCCCCAGATC |
| Rep (382bp)  (-804to -422) | 64 | **GGTACC**GAGTTTGGAGGTGCAGGAGA | **AGATCT**GAAGCAGCGGGCAGGACACACC |

All the forward primers contained KpnI restriction site as 5'overhang while reverse primers contained BglII restriction site as 5' overhang. These restriction sites were added to make the amplicons compatible for ligation into the pGL3 vectors.

**Supplementary Table S5.** ChIP primers used for analyzing *smarcal1* promoter.

| **Amplicon size (bp)** | **Annealing temperature (^o^C)** | **Forward primer (5'→3')** | **Reverse primer (5'→3')** |
| --- | --- | --- | --- |
| *smarcal1* promoter Pair 2 (161bp) | 68 | GATGACTACAATGATGACGATGATGGC | GAGTCGGCTGTCTCCAGAGGACATC |
| *smarcal1* promoterPair 4 (205bp) | 64 | GCGTTGCCTGCTGGGAAATGTAGTTCAG | CACCCAGGGAGGCCTAGAACGCGAGC |
| *smarcal1* promoter Pair 9 (261bp) | 66 | GCCAGACAAATTGGTGTGTCCTGCCCGC | GTAATCTTACCAGCCATCAATAACCACAG |
| *smarcal1* promoter Pair 10 (219bp) | 66 | CTTTATGTAGCATCTTAATTTGCACAGTG | TCCCATAGCCTGCACACTGAATTCTC |

**Supplementary Table S6.** ChIP primers used for analyzing *brg1* promoter.

| **Amplicon size (bp)** | **Annealing temperature (^o^C)** | **Forward primer (5'→3')** | **Reverse primer (5'→3')** |
| --- | --- | --- | --- |
| *brg1* promoter Pair1 (130bp) | 60 | TCAGGGCCTTCACTCTCGGAGGAAG | TGAGGTGAACACTGGGAGGATCATGG |
| *brg1* promoter Pair 2 (187bp) | 69 | CCCAGGCTGGAGTGCAATAGTACG | GGAGATGAGACTATCCTGGCTAACACG |
| *brg1* promoter Pair 5 (239bp) | 60 | TCCGCTCTGCGCATGCTCCGG | CACCGCGGCGGCCTCTCCAGCTTC |

The ChIP primer pairs 2 and 5 were also used for amplifying the primer 2 DNA as well as TSS1 respectively.

**Supplementary Table S7.** Primers used for quantitative real-time RT-PCR.

| Gene | **Forward primer (5'→3')** | **Reverse primer (5'→3')** |
| --- | --- | --- |
| *smarcal1* | TCCCATCTGTTCATTGAATATATCTTGGAC | GCTGCACGTGCTTTCTCTTCAAGCTC |
| *brg1* | GCGAGTACAGGCTGCAGGCT | TGGCGCAGCTGCCTCCTG |
| *Gapdh* | GGTCGGAGTCAACGGATTTGGTC | GAGGGATCTCGCTCCTGGAAG |

**SUPPLEMENTARY FIGURE LEGENDS**

**Supplementary Figure S1. Localization of SMARCAL1 in mammalian cells.** (A). Localization in HeLa and HEK 293 cell lines was monitored using a polyclonal antibody raised against the N-terminus region of SMARCAL1 (red). In this case, the secondary antibody was conjugated to TRITC. (B). Localization in HeLa cell line was also probed using three different monoclonal antibodies-1H10, 6E12, and 10E8- raised against bovine SMARCAL1 (green). The secondary antibody was conjugated to FITC. The scale in these two experiments is 10 µm. (C). Localization of GFP-SMARCAL1 in HeLa cells. The scale in this experiment is 5 µm. Cells were transiently transfected with Lap-Zeo-SMARCAL1 construct and cells were imaged 36 hours post-transfection. In all cases, the nucleus was stained using Hoechst 33342.

**Supplementary Figure S2. HeLa cell cycle analyzed using FACS**. HeLa cells were synchronized as explained in the Supplementary methods and analyzed using FACS at indicated time points.

**Supplementary Figure S3. Quantitation of fluorescence units of confocal images**. The fluorescence units for SMARCAL1, β-tubulin (cytoplasmic marker) and Hoechst (nuclear stain) were quantitated for all time points. The quantification was done using the “intensity line profile” feature of the NIS-Elements AR (Advanced Research) software. Briefly, an arrow was drawn across each cell such that it crossed the nucleus in the center. For each individual channel, pixels along this arrow were shown by the software. The numbers were individually averaged for all the cells and plotted using SigmaPlot.

**Supplementary Figure S4. Doxorubicin treatment induces DNA damage.** DNA damage caused by doxorubicin treatment was assessed using comet assay. (A). Fluorescence images demonstrating the extent of DNA damage in untreated cells (Control), cells treated with 2 µM doxorubicin for 10 minutes, and cells treated with 100µM H_2_O_2_ for 10 min (positive control). Scale - 1000µm. (B). The tail length, tail moment and Olive tail moment was estimated using CASP lab software^3^. The data from 2 independent experiments is represented as a box plot. In each experiment at least 150 cells were analyzed. The units are represented as Arbitrary Units (A.U.)

**Supplementary Figure S5. Analysis of *SMARCAL1* promoter.** (A). Putative promoter architecture of *SMARCAL1* gene and the deletion constructs used in the analysis. (B). The deletion constructs were cloned into the pGL3 basic vector and transiently transfected into HeLa cells. Luciferase activity was measured 36 hours after transfection and normalized with respect to Renilla. (C). The positive regulatory elements (Enh1 and Enh2) and the repressor elements were cloned into pGL3 promoter vector to delineate their function. The constructs were transiently transfected into HeLa cells and assayed for luciferase activity after 36 hours. (D). The positive regulatory elements (Enh1 and Enh2) and repressor elements were cloned into pGL3 basic vector to determine whether they possess any promoter activity. The constructs were transiently transfected into HeLa cells and assayed for luciferase activity after 36 hours. In all these experiments the luciferase activity was normalized with respect to Renilla. (E). Sp1 localization on *smarcal1* promoter was analyzed by ChIP using anti-Sp1 antibody. The presence of Sp1 was detected using a primer pair 10 that encompasses the transcription start site (TSS).

**Supplementary Figure S6. BRG1 binds to *SMARCAL1* promoter.** (A). Depiction of *SMARCAL1* promoter with BRG1, RNAPII, and H3K9Ac localization. (B). The primer pair 2 was used to probe the occupancy of BRG1 on *SMARCAL1* promoter. (C). The primer pair 4 was used to probe the occupancy of BRG1 on *SMARCAL1* promoter. (D). The occupancy of RNAPII and H3K9Ac on *SMARCAL1* promoter was probed using primer pair 4. (E). The occupancy of BRG1 on *SMARCAL1* promoter was probed using primer pair 9. (F). The occupancy of RNAPII and H3K9Ac on *SMARCAL1* promoter was probed using primer pair 9. (G). The occupancy of BRG1 and RNAPII on *SMARCAL1* promoter was probed using primer pair 10. In these experiments untreated HeLa cells were used for ChIP assays. (H). The levels of of BRG1, RNAPII, H3K9Ac, and H3K9(Me)2 bound to *SMARCAL1* promoter was quantitated from the gel pictures (pixel density) using Image J software. (I). Occupancy of BRG1, RNAPII, H3K9Ac, and H3K9(Me)2 on *SMARCAL1* promoter at primers 9 and 10 was analyzed in untreated HeLa cells using quantitative real-time RT-PCR.

**Supplementary Figure S7. BRG1 levels vary as a function of DNA damage.** (A). HeLa cells were treated with 2 µM doxorubicin for 10 minutes and BRG1 levels were compared to the untreated cells by western blot using anti-BRG1 antibody. The quantitation was done using Image J software. (B). The transcript levels of *brg1* in untreated and treated cells were analyzed by quantitative real-time RT-PCR (p value <0.05). (C). Comparison of *smarcal1* and *brg1* transcript levels in untreated and doxorubicin treated cells (p value <0.05). (D). HeLa cells were synchronized using double thymidine block and collected every two hours after release from block. BRG1 levels were analyzed by western blot using polyclonal antibody against the protein. (E). The transcript levels in these cells were analyzed by quantitative real-time RT-PCR. (F). HeLa cells were synchronized using double thymidine block and cells were harvested every two hours after release from the block. Prior to harvesting, the cells were treated for 10 minutes with 2 µM doxorubicin. The levels of BRG1 were analyzed by western blot using polyclonal antibody against the protein. (G). The transcript levels in these cells were analyzed by quantitative real-time RT-PCR. (H). Comparison of the *brg1* transcript levels in untreated and doxorubicin treated synchronized cells. The star indicates significant difference between untreated and doxorubicin treated cells at p values < 0.05 for each of the time point. In all these experiments, GAPDH was used as the internal control. Uncropped western blot images are provided in Supplementary Fig. S12.

**Supplementary Figure S8.** **SMARCAL1 binds to *brg1* promoter.** (A). The architecture of *brg1* promoter with RNAPII and SMARCAL1 localization. ChIP was performed using antibodies against SMARCAL1 and RNAPII to probe their occupancy on *brg1* promoter. Primer pairs 2 and 5 were used in this analysis to map the position of SMARCAL1 and RNAPII on *brg1* promoter. (B). The occupancy of SMARCAL1 and RNAPII on *brg1* promoter was probed using primer pair 2. (C). The occupancy of SMARCAL1 and RNAPII on *brg1* promoter probed using primer pair 5. (D). The levels of of SMARCAL1 and RNAPII bound to *brg1* promoter was quantitated from the gel pictures (Pixel density) using Image J. software. (E). Occupancy of SMARCAL1 and RNAPII was estimated on *brg1* promoter at primer 2 region in untreated HeLa cells using quantitative real-time RT-PCR. (F). Occupancy of SMARCAL1 and RNAPII on *brg1* promoter was estimated at primer 5 region in untreated HeLa cells using quantitative real-time RT-PCR.

**Supplementary Figure S9.** Predicted stem-loop structures for G, C and stem-loop DNA.

**Supplementary Figure S10.**  **The regulatory loop is operative only when both BRG1 and SMARCAL1 are expressed.** (A). Western blot showing the levels of SMARCAL1 and BRG1 in untreated and treated (2 μM doxorubicin; 10 minutes) HepG2 and A549 cells. The bands were quantitated using Image J software and normalized with respect to β-actin. (B) Quantitative real-time RT-PCR data comparing the transcript levels of *brg1* in HeLa, HepG2, and A549 cells before and after treatment with 2 µM doxorubicin for 10 min. Star indicates p value < 0.05. (C). Quantitative real-time RT-PCR data comparing the transcript levels of *SMARCAL1* in HeLa, HepG2, and A549 cells before and after treatment with 2 µM doxorubicin for 10 min. The data was significant at p values <0.05. (D). Quantitative real-time RT-PCR showing the transcript levels of *brg1* and *SMARCAL1* in untreated and treated (100 μM H_2_O_2_; 10 minutes). Uncropped western blots are provided in Supplementary Fig. S13.

**Supplementary Figure S11. Uncropped western blots showing** (A). SMARCAL1 levels in untreated and doxorubicin (2 µM; 10 min) treated HeLa cells. β-actin was used as loading control. (B). SMARCAL1 expression during cell cycle. Cells were blocked using double thymidine block and collected at 0, 4, 8, 12, 14 hours after release from the block. (C). SMARCAL1 expression after treatment with doxorubicin (2 µM; 10 min) during cell cycle. Cells were blocked using double thymidine block and collected at 0, 4, 8, 12, 14 hours after release from the block. Prior to collection the cells were treated with doxorubicin. β-actin was used as loading control.

**Supplementary Figure S12**. **Uncropped western blots showing** (A). BRG1 levels in control and ShA cells. β-actin was used as loading control. (B). BRG1 levels in untreated and doxorubicin (2 µM; 10 min) treated HeLa cells. β-actin was used as loading control. (C). BRG1 expression during cell cycle. Cells were blocked using double thymidine block and collected at 0, 4, 8, 12, 14 hours after release from the block. UB indicates unblocked cells. β-actin was used as loading control. (D). BRG1 expression after treatment with doxorubicin (2 µM; 10 min) during cell cycle. Cells were blocked using double thymidine block and collected at 0, 4, 8, 12, 14 hours after release from the block. Prior to collection the cells were treated with doxorubicin. UB indicates unblocked cells. β-actin was used as loading control.

**Supplementary Figure S13. Uncropped western blots showing** expression of SMARCAL1, BRG1 and β-actin in untreated and treated (2 μM doxorubicin; 10 minutes) A549 and HepG2 cells. The bands were quantitated using Image J software and normalized with respect to β-actin.

**REFERENCES**

1. Kikin, O., D’Antonio, L. & Bagga, P. S. QGRS Mapper: a web-based server for predicting G-quadruplexes in nucleotide sequences. *Nucleic Acids Res.* **34,** W676–W682 (2006).

2. Zuker, M. Mfold web server for nucleic acid folding and hybridization prediction. *Nucleic Acids Res.* **31,** 3406–3415 (2003).

3. Końca, K. *et al.* A cross-platform public domain PC image-analysis program for the comet assay. *Mutat. Res.* **534,** 15–20 (2003).
